# Association of Stanford, DeBakey classification and false-lumen blood flow with age of onset in acute aortic dissection

**DOI:** 10.3389/fcvm.2026.1762060

**Published:** 2026-03-04

**Authors:** Kentaro Shirakura, Ryohei Ushioda, Shingo Kunioka, Jeonga Lee, Hidenobu Akamatsu, Akito Inoue, Yuki Setogawa, Shogo Takahashi, Daisuke Takeyoshi, Kyohei Oyama, Hiroyuki Kamiya, Masahiro Tsutsui

**Affiliations:** 1 Department of Cardiac Surgery, Asahikawa Medical University, Hokkaido, Japan; 2 Department of Cardiovascular Surgery, Asahikawa Red Cross Hospital, Hokkaido, Japan

**Keywords:** AADA, AADB, DeBakey classification, false lumen, Stanford

## Abstract

**Background:**

Younger patients with acute aortic dissection (AAD) are thought to present with distinct anatomical and hemodynamic features compared with older patients. This study evaluated the association between age, dissection extent, and false-lumen flow characteristics in AAD.

**Methods and results:**

We retrospectively analyzed 400 consecutive patients with radiologically confirmed AAD from April 2014 to April 2024, categorizing them into a younger group (<76 years, *n* = 229) and an older group (≥76 years, *n* = 171). The distribution of Stanford type A vs. B dissections was similar between groups. However, younger patients more frequently demonstrated DeBakey type I dissection, whereas older patients more commonly had type II. Regarding false-lumen morphology, younger patients showed a higher prevalence of communicating false lumen, while non-communicating patterns predominated in the elderly. Although overall sex distribution of false-lumen types was not significantly different, younger males were more prevalent within each subtype. Preoperative malperfusion occurred more often in younger patients, whereas 30-day mortality did not differ significantly between age groups.

**Conclusions:**

Younger AAD patients typically exhibit more extensive dissection and patent false-lumen flow, while older patients more often present with localized dissection and thrombosed false lumen. These age-related distinctions may reflect progressive aortic wall stiffening and should be considered in clinical assessment and management strategies.

## Introduction

Acute aortic dissection (AAD) is usually diagnosed in patients aged between 50 and 80 years, and the incidence has been estimated to range from 5 to 30/1 million persons annually worldwide (1). A key pathological feature of AAD is the intimal tear of the vessel wall, allowing blood to enter the media layer, leading to further spread of the tear. This condition carries a high mortality risk, especially during the acute phase. In 1996, the University of Michigan initiated the International Registry of Acute Aortic Dissection (IRAD), which revealed that the mean age of onset is 63.1 years, with approximately 7% of cases occurring in patients under 40 years of age (1). Some research investigated AAD based on age or gender. In general, older patients are thought to be more prone to acute aortic dissection due to a higher incidence of age-related atherosclerosis and stiffening of the thoracic aorta. However, Merkle-Storms et al. hypothesized that younger patients suffer more extensive DeBakey type I dissection due to aortic wall integrity, allowing unhindered extension within the layers. They concluded that extensive DeBakey type I dissections are less common in older patients (≥70 years) compared to younger patients, who are also more likely to present with preoperative organ malperfusion and related complications (2) Another study investigating gender-related differences in type A aortic dissection reported that acute type A aortic dissection (AADA) occurs almost twice as frequently in men as in women. Men more commonly present with stroke, visceral and renal malperfusion, Marfan syndrome, and more extensive aortic lesions. Furthermore, women tend to develop AADA at an older age compared to men (3). Thus, relationship between disease extent in AADA and patients' gender and age has been well studied. However, the relationship between the incidence ratio of Stanford type A and type B aortic dissection and patients' gender and age has been scarcely investigated. Furthermore, few studies have investigated the association between patient gender and age and the incidence of patent vs. thrombosed false lumen types in aortic dissection. In general, prognosis of acute aortic dissection with complete thrombosed false lumen (intramural hematoma) is better than those with patent false lumen (4). In our daily practice, we have an impression that older patients with aortic dissection tend to have thrombosed false lumen. To investigate the relationship between disease extent and behavior of false lumen blood flow patterns in acute aortic dissection and patients' gender and age may help better understanding of acute aortic dissection and improve treatment strategy.

In this study, we examined the association between patients' gender and age and Stanford and DeBakey classifications, as well as false-lumen blood flow patterns, in patients with AAD.

## Methods

**Figure 1:**
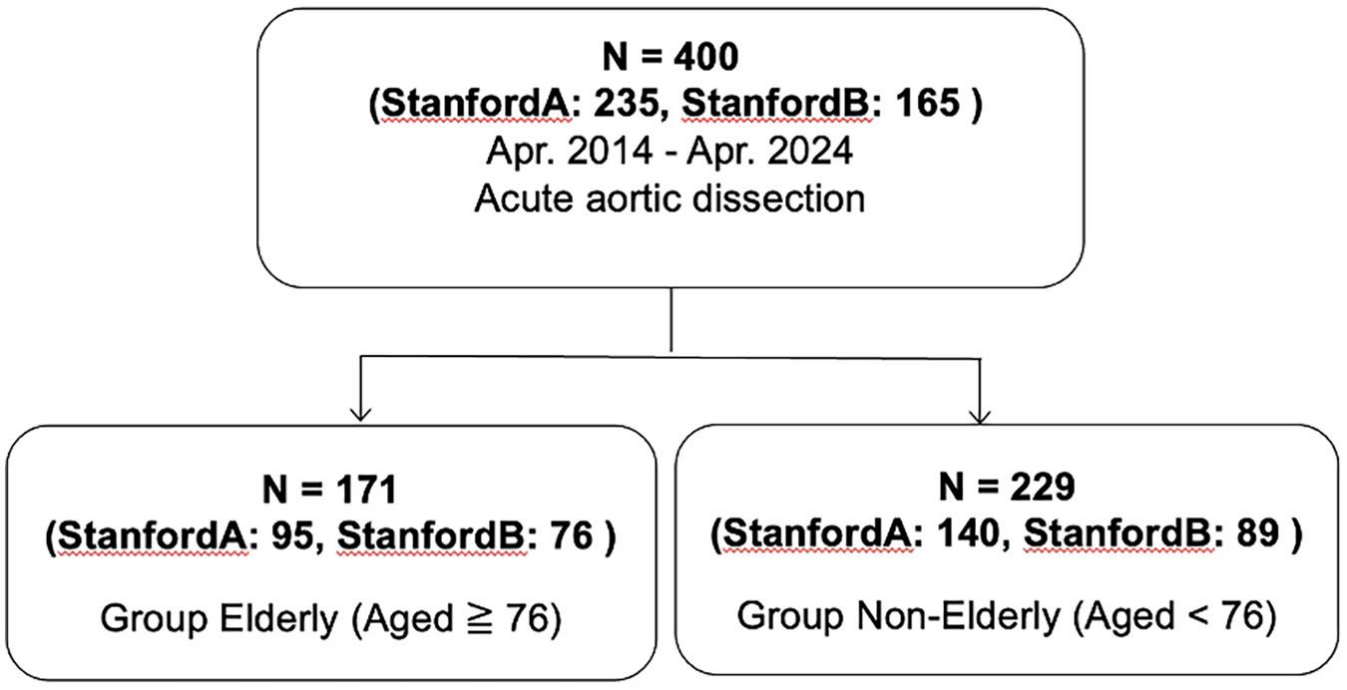
Flowchart of patient selection and group stratification. A total of 400 consecutive patients with radiologically confirmed acute aortic dissection (AAD) were analyzed. After excluding traumatic or iatrogenic cases, patients were stratified into two groups based on a clinically and statistically determined age threshold: the Non-Elderly group (<76 years, *n* = 229) and the Elderly group (≥76 years, *n* = 171).

This study was done retrospectively and was conducted independently at the Asahikawa Medical University. We included 400 patients, at our institute, from April 2014 and April 2024. All patients included in the present study had had a radiologically confirmed diagnosis of AAD. The exclusion criteria were as follows: (1) aortic dissection with an onset of >14 days; (2) traumatic or iatrogenic AAD. In the current study, the association between age of onset, gender and Stanford and DeBakey classifications, as well as false-lumen blood flow patterns, in patients with AAD was examined. The results were compared between patients aged 76 years and older (Group Elderly, *n* = 171) and those younger than 76 years (Group Non-Elderly, *n* = 229) (Figure 1). Detailed justification of grouping regarding age threshold in the present study is described in the results section.

The DeBakey classification was determined based on either intraoperative confirmation of the entry tear or the assumption that ulcer-like projection (ULP)-like changes on contrast-enhanced computed tomography (CT) represented the entry. In cases where no ULP-like changes were observed during follow-up, the classification was provisionally based solely on the site of dissection. Based on the JCS/JSCVS/JATS/JSVS 2020 Guideline on Diagnosis and Treatment of Aortic Aneurysm and Aortic Dissection, intramural hematoma (IMH) was classified as a non-communicating false lumen in terms of false-lumen blood flow patterns (2). Classic dissection and ULPs exceeding 15 mm in the craniocaudal direction within a thrombosed false lumen on contrast-enhanced CT are defined as communicating false lumens. Conversely, ULPs smaller than 15 mm were categorized as ULP-type aortic dissections and were not classified under either communicating or non-communicating false-lumen blood flow patterns. All CT image assessments were performed independently by two board certified cardiovascular surgeons. Any discrepancies in classification were resolved through consensus adjudication with a third senior surgeon; qualitative interobserver agreement for anatomical classifications was consistently high.

### Statistical analysis

Results are expressed as means ± standard deviation. Continuous variables exhibiting a normal distribution were tested using the t-test and continuous variables exhibiting a non-normal distribution were tested using the Mann–Whitney U-test. For categorical variables, Fisher's test was used. Multivariable logistic regression analysis was performed to evaluate the impact of age on clinical outcomes. In these models, age was treated as a continuous variable (per 1-year increment) to maintain statistical power and avoid arbitrary categorization. Categorical covariates were coded as follows: sex (male = 1, female = 0) and hypertension (presence = 1, absence = 0). Missing data were handled using a complete-case approach, and the number of patients included in each model is explicitly reported in the tables. To ensure model stability, multicollinearity was assessed using the variance inflation factor (VIF), with all values confirmed to be below 5.0, indicating no significant collinearity among the independent variables. For the 30-day mortality model, age, sex, and hypertension were selected as primary covariates based on clinical relevance. Notably, treatment strategy (open surgery vs. endovascular vs. conservative) was intentionally excluded from the multivariable model. This decision was made because treatment choice often lies on the causal pathway between age and mortality. Including treatment strategy as a covariate could lead to overadjustment bias, potentially masking the true independent association between age and early survival.

The log-rank test was used to determine the survival rate. Statistical significance was set at *p* < 0.05. Statistical analyses were performed using Stata software (StataCorp, College Station, Texas, USA).

### Ethical standards

This study was performed in accordance with the Declaration of Helsinki and was approved by the Institutional Review Board of Asahikawa Medical University (Approval No. 23005). Given the retrospective nature of the study and the use of anonymized data, the requirement for written informed consent was waived by the board. The opportunity for patients to opt-out was ensured through a notification posted on our institutional website.

### Data availability

The datasets generated during and/or analyzed during the current study are available from the corresponding author upon reasonable request.

## Results

### Demographics and clinical presentation

**Figure 2:**
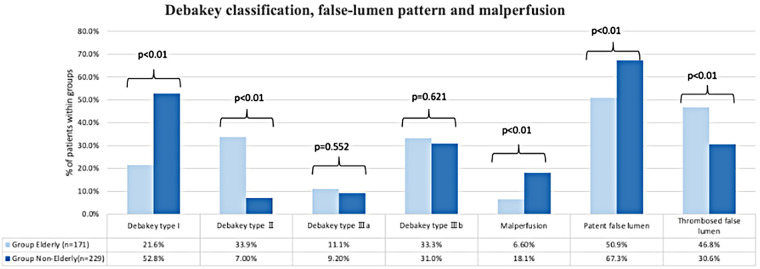
Sex distribution between the Non-Elderly and Elderly groups. The proportion of male patients was significantly higher in the Non-Elderly group (62.0%) compared to the Elderly group (36.3%, *p* < 0.01). Conversely, female patients predominated in the elderly cohort.

Among 400 patients with acute aortic dissection, 235 (58.8%) were Stanford A and 165 (41.3%) were Stanford B, and 241 (60.3%) were communicating false lumen pattern and 150 (37.5%) were non-communicating false lumen pattern. Nine cases were excluded as ULP-type aortic dissections. No significant sex differences were observed in the overall distribution of false lumen patterns (*p* = 0.759). However, in subgroup analyses, the proportion of male patients was consistently higher in the younger age groups, whereas that of female patients was higher in the elderly group. The patients' characteristics are shown in Table 1. The mean age of the patients was 82.6 ± 5.5 years in Group Elderly and 63.2 ± 10.3 years in Group Non-Elderly (*p* < 0.01). The proportion of male patients was 36.3% in Group Elderly and 62.0% in Group Non-Elderly (*p* < 0.01), shown in Figure 2. Regarding comorbidities, the number of cases of chronic renal disease, hemodialysis (HD), chronic obstructive pulmonary disease (COPD), diabetes mellitus (DM), cerebral vascular accident (CVA), steroid use, dyslipidemia (DLP), hypertension (HTN), atrial fibrillation (Af), peripheral vascular disease did not differ between the groups. Regarding clinical presentation, the incidence of malperfusion was significantly higher in Group Non-Elderly compared to Group Elderly (6.4% vs. 17.9%; *p* < 0.01). A detailed breakdown by organ territory revealed that this difference was primarily driven by a higher incidence of renal malperfusion (6.6% vs. 0.6%, *p* < 0.01) and peripheral malperfusion (9.2% vs. 0.6%, *p* < 0.01) in younger patients. Cerebral malperfusion also showed a trend toward a higher prevalence in the non-elderly group, although this difference did not reach statistical significance (6.6% vs. 2.3%, *p* = 0.06). In contrast, no significant age-related differences were observed in the incidence of coronary malperfusion (0.9% vs. 0.6%, *p* = 1.00), spinal malperfusion (1.3% vs. 0.0%, *p* = 0.26), or visceral malperfusion (0.4% vs. 1.2%, *p* = 0.58).

**Table 1:** Patients’ characteristics.

Variable	All patients	Group Elderly	Group Non-Elderly	*P*-value
	*n* = 400	*n* = 171	*n* = 229	
Age, mean ± SD years	71.5 ± 12.5	82.6 ± 5.5	63.2 ± 10.3	<0.01
Male gender, *n* (%)	204 (51)	62 (36.3)	142 (62.0)	<0.01
BMI, kg/m, mean ± SD	23.5 ± 3.5	22.4 ± 3.2	24.6 ± 4.7	0.440
Comorbidity, n(%)				
Chronic renal disease (Cr≧1.5)	47 (11.8)	16 (9.4)	31 (13.5)	0.334
HD	11 (2.9)	6 (3.5)	5 (2.2)	0.539
COPD	9 (2.3)	5 (2.9)	4 (1.7)	0.749
DM	30 (7.5)	21 (12.3)	9 (3.9)	0.232
CVA	3 (0.8)	3 (1.8)	0 (0)	0.278
Steroid use	10 (2.5)	4 (2.3)	6 (2.6)	1.000
DLP	77 (19.3)	34 (19.9)	43 (18.8)	0.797
HTN	71 (17.8)	40 (23.4)	31 (13.5)	0.292
Af	8 (2.0)	5 (2.9)	3 (1.3)	0.895
Peripheral vascular disease	8 (2.0)	5 (2.9)	3 (1.3)	0.895
Clinical presentation, *n* (%)				
Cardiopulmonary resuscitation	11 (2.9)	3 (1.8)	8 (3.5)	0.440
Cardiac tamponade	14 (3.5)	5 (2.9)	9 (3.9)	1.00
Malperfusion	52 (13.1)	11 (6.4)	41 (17.9)	< 0.01
Coronary	3 (0.8)	1 (0.6)	2 (0.9)	1.000
Cerebral	19 (4.8)	4 (2.3)	15 (6.6)	0.06
Spinal	3 (0.8)	0	0	0.26
Visceral	3 (0.8)	2 (1.2)	1 (0.4)	0.58
Renal	16 (4.0)	1 (0.6)	15 (6.6)	<0.01
Peripheral	22 (5.5)	1 (0.6)	21 (9.2)	<0.01

BMI, body mass index; HD, hemodialysis; COPD, chronic obstructive pulmonary disease; DM, diabetes mellitus; CVA, cerebral vascular accident; CAD, coronary artery disease; DLP, dyslipidemia HTN: hypertension, Af: atrial fibrillation.

^※^Categorical variables are reported as *n* (%) and compared using the Chi-square test or Fisher's exact test. Continuous variables are reported as mean ± SD and compared using the t-test.

### Aortic dissection features

**Figure 3:**
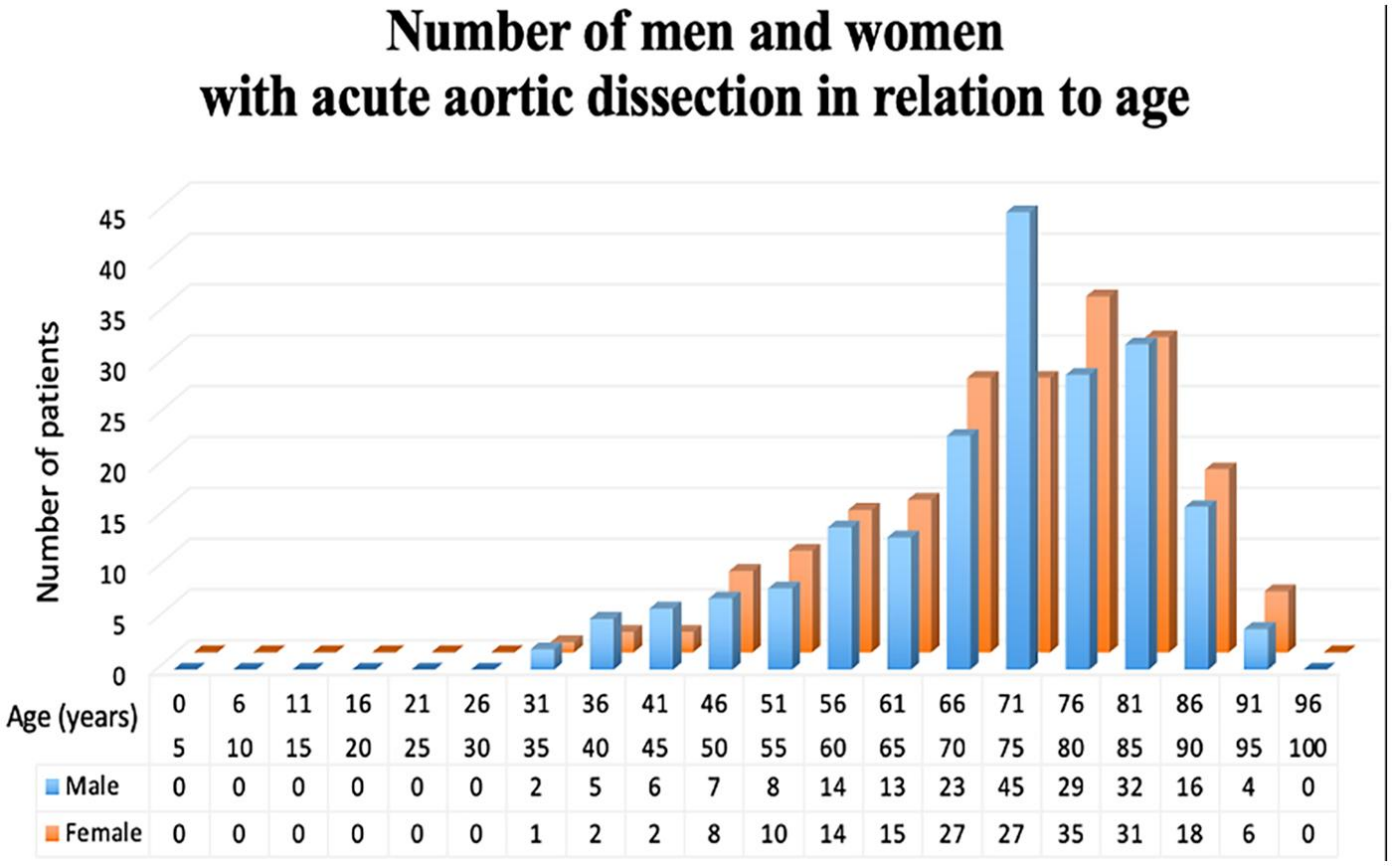
Comparison of DeBakey classification and false-lumen blood flow patterns by age group. Distribution of DeBakey types, showing a significantly higher prevalence of type I in younger patients and type II in older patients (*p* < 0.01). Incidence of false-lumen hemodynamic patterns; communicating false lumens were more frequent in the Non-Elderly group, while non-communicating patterns predominated in the Elderly group (*p* < 0.01).

Dissection-specific data are shown in Table 2. There were no significant differences between the two groups concerning the Stanford classification (Stanford A: 55.6% in Group Elderly vs. 61.1% in Group Non-Elderly; *p* = 0.259, and Stanford B: 44.4% in Group Elderly vs. 38.9% in Group Non-Elderly; *p* = 0.259). DeBakey type I cases were more frequent in Group Non-Elderly (21.6% vs. 52.8%; *p* < 0.01), whereas DeBakey type II cases were more common in Group Elderly (33.9% vs. 7.0%; *p* < 0.01). No significant differences were observed between the groups in DeBakey type IIIa or DeBakey type IIIb. (Figure 3) Regarding false lumen blood flow patterns, communicating false lumen cases were more frequent in Group Non-Elderly (50.9% vs. 67.3%; *p* < 0.01), while non-communicating false lumen cases were more common in Group Elderly (46.8% vs. 30.6%; *p* < 0.01).

**Table 2:** Dissection-specific data.

Variable	All patients	Group Elderly	Group Non-Elderly	*P*-value
	*n* = 400	*n* = 171	*n* = 229	
Stanford classification,n(%)				
Stanford A	235 (58.8)	95 (55.6)	140 (61.1)	0.259
Stanford B	165 (41.3)	76 (44.4)	89 (38.9)	0.259
DeBakey classification,n(%)				
DeBakey type I	158 (39.5)	37 (21.6)	121 (52.8)	<0.01
DeBakey type II	74 (18.5)	58 (33.9)	16 (7.0)	<0.01
DeBakey type IIIa	40 (10.0)	19 (11.1)	21 (9.2)	0.522
DeBakey type IIIb	128 (32.0)	57 (33.3)	71 (31.0)	0.621
False-lumen blood flow patterns,n/N(%)				
Communicating false lumen	241/400 (60.3)	87/171 (50.9)	154/229 (67.3)	<0.01
Male	121/241 (50.2)	28/87 (32.2)	93/154 (60.4)	<0.01
Non-communicating false lumen	150/400 (37.5)	80/171 (46.8)	70/229 (30.6)	<0.01
Male	83/150 (55.3)	34/80 (42.5)	49/70 (70.0)	<0.01

Categorical variables are reported as *n* or n/N (%) and compared using the Chi-square test or Fisher's exact test.

N represents the total number of patients with available data for each specific variable.

To determine the independent predictors of anatomical extent and clinical outcomes, we performed multivariable logistic regression analyses adjusting for age, sex, and hypertension (Table 3). Regarding the longitudinal extent of the dissection, younger age was a robust independent predictor of DeBakey type I lesions (adjusted odds ratio [aOR], 0.947; 95% confidence interval [CI], 0.929–0.965; *p* < 0.01). Hypertension was also independently associated with DeBakey type I lesions (aOR, 3.549; 95% CI, 2.258–5.579; *P* < 0.01), whereas male sex did not reach statistical significance (*p* = 0.17). Similarly, for false-lumen hemodynamic patterns, younger age remained a highly significant independent predictor of a communicating false lumen (aOR, 0.945; 95% CI, 0.926–0.964; *p* < 0.01). In this model, hypertension (aOR, 2.392; 95% CI, 1.539–3.718; *p* < 0.01) and male sex (aOR, 0.593; 95% CI, 0.373–0.941; *p* = 0.03) were also identified as independent factors associated with false-lumen patency. Preoperative malperfusion was significantly associated with both younger age (aOR, 0.946; 95% CI, 0.923–0.969; *p* < 0.01) and male sex (aOR, 0.324; 95% CI, 0.163–0.642; *p* < 0.01). Hypertension also independently contributed to the risk of malperfusion (aOR, 2.128; 95% CI, 1.129–4.014; *p* = 0.02).

**Table 3:** Multivariable logistic regression analysis (adjusted for Sex and hypertension).

Dependent Variable	Factor	Adjusted Odds Ratio	95% Confidence Interval	*P*-value
DeBakey Type I (*n* = 400)	Age	0.947	0.929–0.965	< 0.01
	Sex (Male)	0.72	0.453–1.146	0.17
	Hypertension	3.549	2.258–5.579	<0.01
Communicating False Lumen (*n* = 391)	Age	0.945	0.926–0.964	<0.01
	Sex (Male)	0.593	0.373–0.941	0.03
	Hypertension	2.392	1.539–3.718	<0.01
Preoperative Malperfusion (*n* = 400)	Age	0.946	0.923–0.969	<0.01
	Sex (Male)	0.324	0.163–0.642	<0.01
	Hypertension	2.128	1.129–4.014	0.02
30-day Mortality (*n* = 400)	Age	1.015	0.980–1.052	0.41
	Sex (Male)	0.42	0.173–1.019	0.06
	Hypertension	1.152	0.521–2.548	0.73

Adjusted odds ratios (AORs) and 95% confidence intervals (CIs) were calculated using a multivariable logistic regression model. The model for each dependent variable was simultaneously adjusted for age, sex, and hypertension. AORs represent the effect size of each factor on the respective outcome, with CIs indicating the precision of these estimates.

In contrast, age was not an independent predictor of 30-day mortality (aOR, 1.015; 95% CI, 0.980–1.052; *p* = 0.41). Furthermore, neither male sex (*p* = 0.06) nor hypertension (*p* = 0.73) showed a significant independent association with early mortality.

### Justification of age threshold

**Figure 4:**
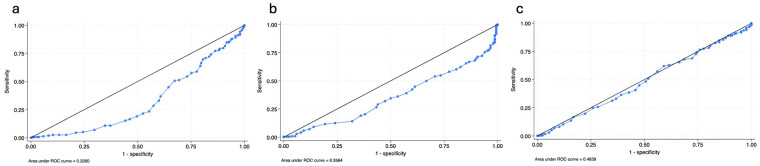
Receiver operating characteristic (ROC) curves of age for **(A)** DeBakey classification, **(B)** false-lumen flow pattern, and **(C)** Stanford classification. The area under the ROC curve (AUC) values are 0.3260 for DeBakey classification, 0.3564 for false-lumen flow pattern, and 0.4839 for Stanford classification. The curves situated below the diagonal reference line indicate that younger age is associated with a higher probability of more extensive dissection (DeBakey type I) and communicating false-lumen patterns. The optimal cut-points were determined using the Youden index.

The primary age threshold of 76 years was selected based on both clinical and statistical considerations. In the Japanese medical system, individuals aged ≥75 years are defined as “late-stage elderly,” which provides an established clinical framework. In addition, receiver operating characteristic (ROC) curve analyses were performed to identify the optimal age cut-off for the study outcomes. The Youden index was used to determine the threshold that maximized the sum of sensitivity and specificity. ROC analyses were conducted for DeBakey classification, false-lumen flow patterns, and Stanford classification (Figure 4). ROC curve analysis demonstrated a significant association between age and the longitudinal extent of dissection. For DeBakey classification, the area under the curve (AUC) was 0.674, indicating moderate discriminative ability. The Youden index identified 76 years as the statistically optimal cut-point (J = 31.95), which was concordant with the predefined clinical threshold.

For false-lumen flow patterns, the AUC was 0.640, with an optimal cut-point of 68 years. Notably, when applying the 76-year threshold, meaningful discriminative performance was preserved. In contrast, age showed no predictive value for Stanford classification (AUC = 0.484), indicating no association between age and the initial site of the intimal tear. To evaluate the robustness of the findings and to exclude threshold dependency, sensitivity analyses were performed using alternative age cut-offs of 70, 75, and 80 years. Across all tested thresholds, younger patients consistently exhibited a higher prevalence of DeBakey type I dissection and communicating false-lumen patterns, with all comparisons reaching statistical significance (all *P* < 0.01). The magnitude and direction of these associations remained consistent, supporting the stability of the observed age-related differences. Detailed results of the sensitivity analyses are summarized in Table 4. Although the ROC-derived optimal age cut-point varied by outcome, we retained 76 years as the primary stratification because it represents a clinically established threshold in the Japanese healthcare system and coincides with the statistically optimal cut-point for DeBakey classification, which reflects the longitudinal extent of dissection. Importantly, the sensitivity analyses using adjacent thresholds demonstrated consistent results, indicating that the study conclusions are robust and not dependent on a single arbitrary age cut-off.

**Table 4:** Sensitivity analysis.

Age Threshold	DeBakey Type I (%)	*P*-value	Communicating false lumen (%)	*P*-value
<70 vs. ≥70	56.3% vs. 29.3%	<0.01	72.2% vs. 53.0%	<0.01
<75 vs. ≥75	54.2% vs. 22.3%	<0.01	68.1% vs. 51.1%	<0.01
<76 vs. ≥76 (Main)	52.8% vs. 21.6%	<0.01	67.3% vs. 50.9%	<0.01
<80 vs. ≥80	47.6% vs. 22.9%	<0.01	65.8% vs. 48.9%	<0.01

Sensitivity analyses of age thresholds (70, 75, 76, and 80 years) for DeBakey type I dissection and communicating false-lumen patterns.

### Patients' therapy and postoperative outcomes

Patients' therapy and 30-day mortality are shown in Table 5. For patients with type A aortic dissection, in the Group Elderly, 90.5% underwent open surgical repair, whereas 4.2% underwent endovascular surgery for closure of the primary entry tear in the descending aorta in patients with DeBakey type IIIb retrograde dissection and 9.5% were managed conservatively. Among the patients initially managed conservatively, four cases in type A aortic dissection eventually required intervention due to disease progression (2 cases converted to open surgery and 2 cases to TEVAR). The primary reasons for conservative management in this group included four cases of high operative risk, as well as four cases of thrombosed false lumen with a small residual false lumen thickness.

**Table 5:** Patients’ therapy and 30-day mortality.

Stanford type	Treatment	Group Elderly	Group Non-Elderly	*P*-value	30-day mortality, n/N (%)	Group Elderly	Group Non-Elderly	*P*-value
		*n* = 95	*n* = 140					
Type A (235)	Open surgery, n/N (%)	86/95 (90.5)	137/140 (97.9)	0.02		7/86 (8.1)	14/137 (10.2)	0.65
	Endovascular surgery, n/N(%)	4/95 (4.2)	1/140 (0.7)	0.16		0	0	N/A
	Conservative, n/N(%)	9/95 (9.5)	2/140 (1.4)	0.02		0	0	N/A
	Urgent, n/N(%)	5/95 (5.4)	6/140 (4.3)	0.49		0	2/6 (33.3)	0.46
	Emergent, n/N(%)	81/95 (85.3)	132/140 (94.3)	0.06		7/81 (8.6)	12/132 (9.1)	1.00
Stanford type	Treatment	Elderly	Non-Elderly	*P*-value				
		*n* = 76	*n* = 89					
Type B (165)	Open surgery, n/N(%)	0	0	N/A		0	0	N/A
	Endovascular surgery, n/N(%)	8/76 (10.5)	8/89 (8.9)	1.00		0	0	N/A
	Conservative, n/N(%)	74/76 (97.4)	85/89 (95.5)	0.83		5/74 (6.8)	1/85 (1.2)	0.10
	Urgent, n/N(%)	1/76 (1.3)	2/89 (2.2)	1.00		0	0	N/A
	Emergent, n/N(%)	1/76 (1.3)	2/89 (2.2)	1.00		0	0	N/A

Categorical variables are reported as n/N (%) and compared using the Chi-square test or Fisher’s exact test.

N represents the total number of patients with available data for each specific variable.

In contrast, in the Group Non-Elderly, 97.9% received open surgery, 0.7% underwent endovascular surgery for closure of the primary entry tear in the descending aorta in patients with DeBakey type IIIb retrograde dissection and 1.4% were treated conservatively, with the main reason for conservative management being thrombosed false lumen with a small residual false lumen thickness.

For patients with type B aortic dissection, in the Group Elderly, 10.5% were treated with endovascular surgery, and 97.4% were managed conservatively, 1.3% were managed urgently and 1.3% were operated emergently. Six cases in type B aortic dissection initially managed conservatively eventually underwent crossover to TEVAR.

The indications for urgent intervention included enlargement of the false lumen, while emergent cases were defined by descending aortic rupture or malperfusion of the left renal artery. In the Non-Elderly Group, 8.9% underwent endovascular repair, 95.5% were managed conservatively, 2.2% required urgent intervention, and 2.2% underwent emergent surgery. Four cases initially managed conservatively eventually underwent crossover to TEVAR in this subtype. The urgent cases were indicated by enlargement of the false lumen, whereas the two emergent cases consisted of descending aortic rupture and left renal artery malperfusion.

As for the patients' therapy of AADA, the cases of open surgery were significantly higher in the Group Non-Elderly (90.5% vs. 97.9%; *P* = 0.02) and the cases of conservative management were significantly higher in the Group Elderly (9.5% vs. 1.4%; *P* = 0.02). Regarding the patients' therapy of AADB, there were no significant differences.

**Figure 5:**
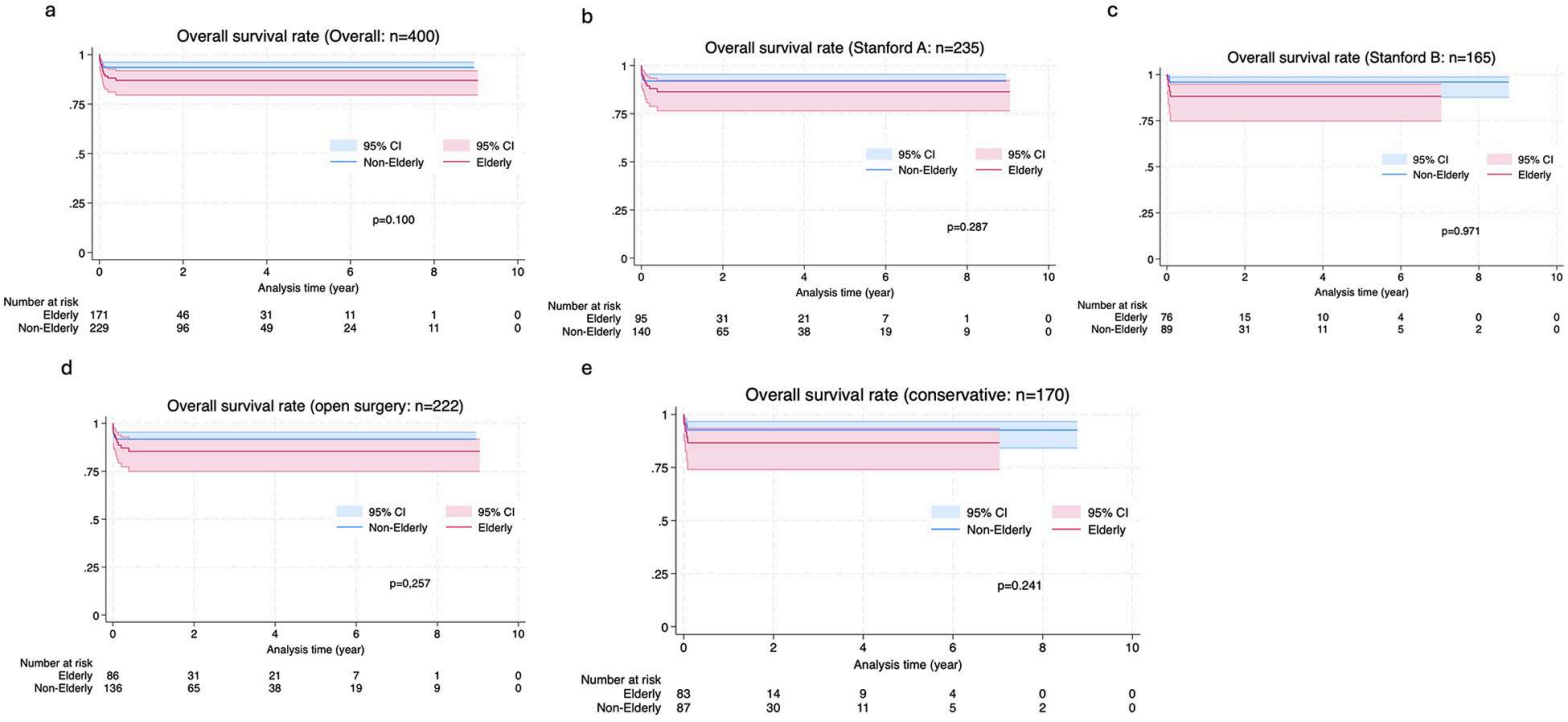
Kaplan-Meier analysis of overall survival. Survival curves are shown for **(A)** the entire cohort (*n* = 400), **(B)** patients with Stanford type A dissection (*n* = 235), **(C)** patients with Stanford type B dissection (*n* = 165), **(D)** patients who underwent open surgical repair (*n* = 222), and **(E)** patients managed conservatively (*n* = 170). Blue lines and red lines represent the Non-Elderly and Elderly groups, respectively, with shaded areas indicating 95% confidence intervals. There were no statistically significant differences in survival between the two age groups in any category: **(A)** P = 0.100, **(B)** P = 0.287, **(C)** P = 0.971, **(D)** P = 0.257, and **(E)** P = 0.241. Notably, the median follow-up duration was significantly shorter in the Elderly group compared to the Non-Elderly group across all cohorts. Numbers at risk are provided below each plot.

Figure 5 illustrates the 10-year overall survival rates for the entire cohort and subgroups.

The median follow-up duration for the entire cohort (*n* = 400) was significantly shorter in the Elderly group than in the Non-elderly group (85 days [IQR, 15–824] vs. 488 days [IQR, 30–1,282]; *p* < 0.01). This significantly shorter follow-up in the Elderly group was consistently observed across subgroups, including patients with Stanford type A dissection (*p* = 0.04), Stanford type B dissection (*p* < 0.01), and those managed with conservative treatment (*p* < 0.01). Kaplan–Meier survival analysis demonstrated no statistically significant difference in overall survival between the Elderly and Non-elderly groups in the total cohort (*p* = 0.100). Similarly, no significant differences in survival were observed between the two age groups among patients with Stanford type A dissection (*p* = 0.287) or Stanford type B dissection (*p* = 0.971). Among patients who underwent open surgery (*n* = 222), overall survival did not differ significantly between the Elderly and Non-elderly groups (*p* = 0.257). Likewise, in patients managed conservatively (*n* = 170), no significant age-related difference in survival was observed (*p* = 0.241). While the log-rank test showed no significant difference in long-term survival (*p* = 0.10), these results should be interpreted cautiously as the number of patients at risk in the Elderly group decreased substantially after the 5-year mark.

A 30-day mortality showed no significant differences between two groups in any classified case.

### Intraoperative variables of open surgery

Intraoperative data of open surgery is shown in Table 6. Eighty-six of 95 patients (90.5%) in the Elderly Group and 137 of 140 patients (97.9%) in the Non-Elderly Group underwent open surgery. In younger patients, a valve-sparing aortic replacement (0% vs. 5.9%; *P* = 0.024) was performed significantly more often in the younger cohort. Older patients were treated significantly more often with hemiarch procedure (80.2% vs. 51.5%; *P* < 0.01), while younger patients underwent total arch replacement more frequently (12.8% vs. 43.4%; *P* < 0.01). No differences were found between the young and the elderly regarding the cannulation sites and selective cerebral perfusion. The overall times for cardiopulmonary bypass (CPB) (167.3 ± 70.9 vs. 192.8 ± 95.4 min; *P* = 0.043) was significantly longer in the younger study group.

**Table 6:** Intraoperative data (open surgery).

Variable	All patients (Open surgery)	Group Elderly	Group Non-Elderly	*P*-value
	*n* = 223	*n* = 86	*n* = 137	
Aortic valve replacement	2/223 (0.9)	2/86 (2.3)	0	0.149
Bentall	6/223 (2.7)	4/86 (4.7)	2/137 (1.5)	0.210
David	8/223 (3.60)	0	8/137 (5.9)	0.024
Yacoub	1/223 (0.5)	0	1/137 (0.7)	1.000
Hemiarch replacement	139/223 (62.6)	69/86 (80.2)	70/137 (51.5)	< 0.01
Partialarch replacement	12/223 (5.4)	7/86 (8.1)	5/137 (3.7)	0.222
Total arch replacement	70/223 (31.5)	11/86 (12.8)	59/137 (43.4)	< 0.01
Elephant trunk	13/223 (5.9)	1/86 (1.2)	12/137 (8.8)	0.018
Frozen elephant trunk	47/223 (21.2)	7/86 (8.1)	40/137 (29.1)	< 0.01
CABG	7/223 (3.2)	1/86 (1.2)	6/137 (4.4)	0.253
Cannulation sites				
Axillary	7/223 (3.2)	3/86 (3.5)	4/137 (3.0)	1.000
Ascending/ arch	50/223 (22.6)	22/86 (25.6)	28/137 (20.7)	0.414
Femoral	167/223 (76.3)	63/86 (74.1)	104/137 (77.6)	0.626
Selective cerebral perfusion				
Antegrade	215/223 (97.3)	83/86 (96.5)	132/137 (97.8)	0.680
Retrograde	50/223 (22.6)	17/86 (19.8)	33/137 (24.4)	0.510
Lowest temperature(℃), mean ± SD	26.4 ± 1.66	26.5 ± 1.66	26.3 ± 1.66	0.173
CPB time(min), mean ± SD	183.2 ± 87.6	167.3 ± 70.9	192.8 ± 95.4	0.043
Cross-clamp time (min), mean ± SD	113.3 ± 55.0	107.5 ± 38.6	116.9 ± 62.9	0.671

CPB, cardiopulmonary bypass.

Categorical variables are reported as **n/N (%)**. The denominator **N** represents the total number of patients who underwent open surgery (*n* = 223 total; *n* = 86 Elderly, *n* = 137 Non-elderly).

Continuous variables are reported as mean ± standard deviation and compared using the Student's t-test.

### Intraoperative variables of endovascular surgery

Intraoperative data of endovascular surgery are summarized in Table 7. Twelve of 95 patients (12.6%) in the Elderly Group and 9 of 140 patients (6.4%) in the Non-Elderly Group underwent endovascular surgery. There were no significant differences in Stanford classification. No differences were observed between the elderly and non-elderly groups regarding the indication for the procedure, type of procedure, or landing zone. Neither operation time (161.9 ± 103.3 vs. 161.7 ± 102.1; *P* = 1.00) nor aneurysm diameter (52.5 ± 9.6 vs. 54.3 ± 14.6) demonstrated significant differences.

**Table 7:** Intraoperative data (endovascular surgery).

Variable	All patients (Endovascular surgery)	Group Elderly	Group Non-Elderly	*P*-value
	*n* = 21	*n* = 12	*n* = 9	
Stanford classification				
Stanford A	5/21 (23.8)	4/12 (33.3)	1/9 (11.1)	0.34
Stanford B	16/21 (76.2)	8/12 (66.7)	8/9 (88.9)	0.35
Indication of procedure				
Malperfusion	2/21 (9.5)	1/12 (8.3)	1/9 (11.1)	1.00
Impending to rupture	1/21 (4.8)	0	1/9 (11.1)	0.43
Progressive enlargement	18/21 (85.7)	10/12 (83.3)	8/9 (88.9)	1.00
Type of procedure				
Standard TEVAR	11/21 (52.3)	5/12 (41.7)	6/9 (66.7)	0.39
Hybrid TEVAR	10/21 (47.6)	6/12 (50.0)	4/9 (44.4)	1
LCCA to LSCA	3/21 (14.3)	1/12 (8.3)	2/9 (22.2)	0.55
RSCA to LSCA	7/21 (33.3)	5/12 (41.7)	2/9 (22.2)	0.64
Landing zone				
Zone 0	1/21 (4.8)	1/12 (8.3)	0	1.00
Zone 1	0	0	0	N/A
Zone 2	10/21 (47.6)	5/12 (41.7)	5/9 (55.6)	0.67
Zone 3	2/21 (9.5)	1/12 (8.3)	1/9 (11.1)	1.00
Zone 4	8/21 (38.1)	4/12 (33.3)	4/9 (44.4)	0.67
Operation time, mean ± SD min	161.8 ± 100.1	161.9 ± 103.3	161.7 ± 102.1	1.00
Aneurysm diameter, mean ± SD mm	53.3 ± 11.9	52.5 ± 9.6	54.3 ± 14.6	0.75

TEVAR, thoracic endovascular aortic repair; LCCA, left common carotid artery; LSCA, left subclavian artery; RSCA, right subclavian artery.

Categorical variables are expressed as n/N (%) and compared using **Fisher’s exact test** due to the small sample size in each cell.

Continuous variables are expressed as mean ± standard deviation and compared using the Student’s t-test.

Denominators: Percentages and *p*-values were calculated based on the number of patients who underwent endovascular surgery in each group (Elderly: *n* = 12, Non-elderly: *n* = 9; Total: *n* = 21).

## Discussion

Despite ongoing advancements in perioperative management for patients with AAD, postoperative morbidity and mortality rates remain high. Outcomes are heavily influenced by the patient's preoperative condition and the expertise of the surgical team and center (5, 6).

This clinical study aimed to evaluate the association between age of onset and Stanford and DeBakey classifications, as well as false-lumen blood flow patterns, in patients with AAD.

Overall, 400 patients with AAD were identified and subsequently analyzed with the following results:

Type of Stanford classification showed no significant differences between two groups.
DeBakey type I aortic dissection was significantly more frequent in younger patients (<76 years), whereas DeBakey type II aortic dissection was significantly more often in older patients (≥76 years).
Younger patients showed significantly higher incidences of communicating false lumen, and older patients showed significantly higher incidences of non-communicating false lumen
No significant sex differences were observed in the overall comparison of false lumen patterns, however the proportion of male patients was consistently higher in the younger groups for both communicating and non-communicating types.
The overall 30-day mortality and long-term survival rate were not significantly different.

### Age-related outcomes

In general, advanced age has been associated with an increased incidence of acute aortic dissection, which has been attributed to age-related changes in the aortic wall, including atherosclerosis and progressive stiffening of the thoracic aorta. Previous studies have reported that older age is an independent risk factor for thoracic aortic dissection, potentially reflecting reduced elasticity and altered biomechanical properties of the aortic wall (2, 3). These age-related changes include medial degeneration, loss of elastic fibers, collagen remodeling, and increased wall thickness, as demonstrated in histopathological studies (7–11). Merkle-Storms et al. revealed that younger patients suffer more extensive DeBakey type I dissection due to aortic wall integrity, allowing unhindered extension within the layers. In this report patients younger than 69 years, aortic dissection more frequently extended into the supra-aortic and propagated significantly further along the aorta compared to older patients. As a result, younger individuals exhibited significantly higher rates of preoperative malperfusion syndromes. In contrast, patients aged 70 years or older more frequently exhibited dissection confined to the ascending aorta. Whereas no significant difference was found regarding 30-day mortality. They concluded that extensive DeBakey type I dissections are less common in older patients (≥70 years) compared to younger patients, who are also more likely to present with preoperative organ malperfusion and related complications (2).

In the present study, we had similar results with the study above. Despite the potential connection favoring the onset of acute aortic dissection, the elderly group more frequently experienced DeBakey type II dissection, which is less extensive than DeBakey type I dissection, compared to younger patients. In contrast, DeBakey type I aortic dissection was significantly more frequent in younger patients (< 76 years). Younger group exhibited significantly higher rates of preoperative malperfusion syndromes. Furthermore no significant difference was found regarding 30-day mortality. Based on these findings, younger patients with acute aortic dissection appear to present with more extensive anatomical involvement. However, the underlying biomechanical mechanisms remain speculative, as direct measures of aortic wall stiffness, calcification burden, or histological degeneration were not available in the present study. Further investigations incorporating quantitative imaging or tissue-based assessments are warranted to clarify these age-related differences.

### Gender-related outcomes

The German Registry of Acute Aortic Dissection Type A (GERAADA) and The International Registry of Acute Aortic Dissections (IRAD) have identified significant gender differences in the incidence of type A aortic dissection. Less than 25% of type

A dissection patients younger than age 40 years were women, and the cut-off age for male dominance was 75 years (3, 12, 13). Some other studies on gender-related differences in patients with acute type A aortic dissection found that women develop the condition at an older age than men. Additionally, men are more likely to present with stroke, visceral and renal malperfusion, and Marfan syndrome. Aortic dissection involving the entire aorta is also more frequent in men. Furthermore, that study observed an increased proportion of female patients aged 75 and older, which aligns with our findings (13–15). These results suggest that both age and gender may influence the clinical presentation of acute type A aortic dissection. Even in our study, in the older age group, the proportion of female patients was significantly higher, whereas in the younger age group, the proportion of male patients was significantly higher. The finding that extensive lesions were more prevalent in the younger cohort is largely consistent with previous studies. These results suggest that not only age but also sex itself may represent an influential factor in the outcomes observed in the present study.

However, the pathomechanisms influencing the extension of aortic dissection within the aortic wall layers are not fully understood. A deeper understanding of the metabolic and biomechanical properties of the aorta is essential to uncover the various factors contributing to aortic dissection development.

### Age-related differences in false lumen blood flow patterns

With regard to false-lumen blood flow patterns, to the best of our knowledge, there have been very few previous studies. In our study, younger patients (< 76 years) exhibited a significantly higher incidence of communicating false lumen patterns, while older patients (≥76 years) showed a higher incidence of non-communicating false lumen patterns. Regarding sex differences, although no significant differences were observed in the overall comparison of false lumen patterns, the proportion of male patients was consistently higher in the younger groups for both communicating and non-communicating types.

According to the data from the IRAD, patients with IMH were generally older than those with classic aortic dissection (mean age 68.7 vs. 61.7 years; *P* < 0.001), showing similar consequences with our study results (1). These findings indicate that the risk of aortic intimal-medial tearing may be generally lower in younger individuals, however when dissection does occur, it tends to progress more rapidly and powerfully. In our hypothesis, the presence of aortic calcification, such as in atherosclerosis, appears to mitigate the risk of undamped extension within the layers of the distal aorta. This phenomenon may partially explain the lower incidence of DeBakey type I aortic dissection observed in older patients as well as the higher incidence of communicating false lumen patterns in younger patients.

### Age-related differences in surgical techniques for type A aortic dissection

Age has been shown to be a strong critical factor of outcomes in previous studies, while treatment strategies and surgical methods differentiate according to age stratification (16, 17).

The study constructed and validated a risk model for predicting operative mortality after AADA surgery including age and other variables, in which older age significantly contributed to an increased risk of mortality, and found extensive aortic repair is associated with significantly higher risk of operative mortality than proximal repair when the predicted probability exceeded a certain threshold. These findings suggested that some high-risk patients, such as the aged individuals, are more likely to benefit from proximal repair rather than extensive repair (17). In our study, aged populations more likely to undergo proximal repair due to less extensive lesions. When it comes to comparison of mortality, generally, the preoperative risk and mortality are expected to be higher in the elderly group; however, no significant differences were observed between the two groups. A potential explanation for this is that, as previously discussed, the younger group often presented with more extensive lesions caused by aortic dissection, a higher prevalence of communicating false lumens, and complications such as malperfusion. Further clinical studies with a larger group of patients might be required to investigate in detail of this field.

### Association between age and surgical intervention in type B aortic dissection

According to current guidelines, uncomplicated type B aortic dissections are generally managed conservatively, whereas cases with complications are recommended to be treated with a stent graft as the first-line strategy. In the present study, the proportion of patients undergoing stent graft repair did not differ between the Elderly and Non-Elderly Groups with type B dissection. This finding suggests that the incidence of complications, such as malperfusion, does not substantially differ by age in patients with type B dissection.

### Limitations

There are several limitations to the present study. First, this was nonrandomized and retrospective study of a single center's experience with a relatively small sample size. Therefore, there may persist a certain degree of selection bias and potential confounding which could have influenced our findings. Second, there was a significant imbalance in the follow-up duration between the two age groups, with the Elderly group having a shorter median follow-up. This discrepancy may be attributed to a higher incidence of non-aortic related background mortality (competing risks) in older patients and administrative censoring resulting from the fixed study period. Consequently, long-term survival comparisons between the groups should be interpreted with caution, particularly in the later time windows where the number of patients at risk becomes small. Such a decrease in the at-risk population can increase the volatility of survival estimates and reduce the statistical power for detecting differences in late outcomes.

Finally, some cases were not included in our analysis due to patient death before arrival at our hospital. This limitation may have influenced the reported results.

## Conclusion

In younger patients, aortic dissection tends to be more extensive, with a higher prevalence of communicating false lumen patterns. Furthermore, they are significantly more likely to present with organ malperfusion and its associated complications. These differences are likely explained by age-related histological changes in the aortic wall, such as increased atherosclerosis in older patients, which may confer greater structural rigidity and resistance to dissection propagation. Nevertheless, the overall 30-day mortality and long-term survival rates did not differ significantly between the groups. The mechanisms governing the extension of aortic dissection within the aortic wall remain incompletely understood. Gaining deeper insights into the aorta's metabolic and biomechanical properties is crucial for identifying the factors that contribute to the progression and development of aortic dissection.

## Data Availability

The raw data supporting the conclusions of this article will be made available by the authors, without undue reservation.
